# Role of TGF-*β* in breast cancer bone metastases

**DOI:** 10.4236/abb.2013.410A4003

**Published:** 2013-10-13

**Authors:** Antonella Chiechi, David L. Waning, Keith R. Stayrook, Jeroen T. Buijs, Theresa A. Guise, Khalid S. Mohammad

**Affiliations:** 1 Division of Endocrinology, Department of Internal Medicine, Indiana University, Indianapolis, USA; 2 Department of Urology, Medical Center, Leiden University, Leiden, The Netherlands

**Keywords:** Transforming Growth Factor-Beta, TGF-*β*, Breast Cancer, Bone Metastasis, Bone, Small Molecule Inhibitors, Antibodies, Bone Resorption

## Abstract

Breast cancer is the most prevalent cancer among females worldwide leading to approximately 350,000 deaths each year. It has long been known that cancers preferentially metastasize to particular organs, and bone metastases occur in ~70% of patients with advanced breast cancer. Breast cancer bone metastases are predominantly osteolytic and accompanied by increased fracture risk, pain, nerve compression and hypercalcemia, causing severe morbidity. In the bone matrix, transforming growth factor-*β* (TGF-*β*) is one of the most abundant growth factors, which is released in active form upon tumor-induced osteoclastic bone resorption. TGF-*β*, in turn, stimulates bone metastatic tumor cells to secrete factors that further drive osteolytic bone destruction adjacent to the tumor. Thus, TGF-*β* is a crucial factor responsible for driving the feed-forward vicious cycle of cancer growth in bone. Moreover, TGF-*β* activates epithelial-to-mesenchymal transition, increases tumor cell invasiveness and angiogenesis and induces immunosuppression. Blocking the TGF-*β* signaling pathway to interrupt this vicious cycle between breast cancer and bone offers a promising target for therapeutic intervention to decrease skeletal metastasis. This review will describe the role of TGF-*β* in breast cancer and bone metastasis, and pre-clinical and clinical data will be evaluated for the potential use of TGF-*β* inhibitors in clinical practice to treat breast cancer bone metastases.

## 1. INTRODUCTION

Cancer is the leading cause of death in economically developed countries. Breast cancer is the most frequently diagnosed cancer in females accounting for 23% (1.38 million) of the total new cancer cases and is the leading cause of cancer death among females worldwide, [1,2]. It is estimated that there are nearly 3 million women living in the United States with a history of invasive breast cancer and it is estimated that over 226,870 new cases of invasive breast cancer were diagnosed in 2012 [3]. Breast cancer frequently metastasizes to the skeleton, and approximately 70% of patients with advanced breast will develop bone metastases [4–6].

Patients with bone metastases are at risk of skeletal complications, including spinal cord compression, pain, pathological fracture, hypercalcemia, complications due to surgery to bone, and radiation therapy. These comorbidities are known collectively as skeletal-related events (SREs). SREs are associated with impaired mobility, reduced quality of life, increased mortality, and higher healthcare costs [7]. Standard antiresorptive treatments decrease skeletal morbidity and delay skeletal related events (SRE), but do not cause regression or cure the disease [6,8]. Cancer patients who develop bone metastases, particularly those with breast and prostate cancer, can survive for many years after diagnosis, during which they will suffer significant morbidity. That is why better treatments are needed to achieve the long-term goal of preventing or curing bone metastases.

The bone microenvironment is unique and provides fertile soil for cancers to thrive. Many growth factors and cytokines are embedded in the mineralized bone matrix and are released during osteoclastic bone resorption. Transforming growth factor-*β* (TGF-*β*) is the most abundant of these factors. The TGF-*β* superfamily also includes other factors involved in bone homeostasis including: activins, inhibins, and bone morphogeneticproteins (BMPs). TGF-*β* that is released from bone is activated by either proteolytic cleavage, interaction with integrins, or pH changes in the local microenvironment [9]. In addition, TGF-*β* stimulates tumor production of pre-osteolytic and osteolytic factors that stimulate further bone resorption [10,11]. This categorizes TGF-*β* as an important factor responsible for driving the feed-forward vicious cycle of tumor growth in bone. Therefore blocking TGF-*β* release, its production and/or signaling is a promising strategy to treat bone metastasis. Over the past several years, several therapeutic strategies have been developed to inhibit TGF-*β*, including, TGF-*β* receptor kinase inhibitors, TGF-*β* neutralizing antibodies, soluble receptor decoys (Fc fusions) and TGF-*β* antisense oligonucleotides [12]. Many of these are now in early-stage clinical trials for various disease indications with particular emphasis as potential cancer therapies, including bone metastases. In this review, we will focus on the role of TGF-*β* in breast cancer and bone metastasis and discuss the potential use of novel TGF-*β* inhibiting compounds and biologics in clinical practice to treat bone metastases.

## 2. TGF-*β* STRUCTURE AND SIGNALING

### 2.1. TGF-*β* Structure

TGF-*β* was originally named for its ability to induce malignant behavior of normal fibroblasts. It is ubiquitously expressed in normal developing and adult tissues. It is a multifunctional cytokine that controls tissue homeostasis by regulating cellular processes such as apoptosis, proliferation and differentiation [13]. TGF-*β* orchestrates the response to tissue injury and mediates repair by inducing epithelial-to-mesenchymal transition (EMT) and cell migration, and it is a critical regulator of the immune response. Dysregulation of TGF-*β* functions have been associated with many disorders, including chronic fibrosis, cardiovascular diseases and cancer [14,15].

The TGF-*β* superfamily includes more than 30 protein ligands divided into subfamilies based on sequence similarity and function. Members of the TGF-*β* superfamily are TGF-*β*s, bone morphogenetic proteins (BMPs), activins, inhibins, growth and differentiation factors (GDFs), NODAL and anti-Müllerian hormone (AMH) [16–18]. The ligands are all synthesized as precursors with a large N-terminal pro-domain necessary for the correct protein folding and dimerization. After cleavage, the mature ligands form homodimers or heterodimers held together by disulfide bonds. In some cases, the prodomain is still associated with the mature protein after secretion via a non-covalent association. TGF-*β* is secreted as a latent precursor: After secretion the pro-domain (latency associated protein, LAP) binds and inactivate the ligand, allowing its association with inhibitory latent TGF binding proteins (LTBPs) that target the complex to the ECM where the latent TGF-*β* is sequestered. In humans, three isoforms of TGF-*β* have been described, TGF-*β*1, TGF-*β*2 and TGF-*β*3. The signaling of these three isoforms is comparable but their expression level differs across tissue types [19]. Signaling mediated by TGF-*β* ligands is transduced through cell surface recaptor complexes of two distinct types of transmembrane serine-threonine kinases, the type I and type II receptors. Seven type I receptors (Activin-recaptor like kinases, ALKs, 1–7) and five type II receptors are known in vertebrates. The ligand binds a type II receptor, which phosphorylates a partner type I receptor, which in turn propagates the signal inside the cell via phosphorylation of downstream Smad-dependent and -independent processes [20].

### 2.2. Smad-Mediated Signaling

In vertebrates, eight Smad proteins are known (Smad 1–8). Smads 1, 2, 3, 5 and 8 are the receptor-associated Smads or R-Smads. While Smad1/5/8 are phosphorylated by ALK1/2/3/6 upon BMP or GDF activation, Smad2/3 are phosphorylated by ALK4/5/7 following TGF-*β*, NODAL or Activin signaling [21]. Active TGF-*β* binds TGF-*β* receptor type II (T*β*RII), which recruits and activates ALK5. ALK5 phosphorylates R-Smad2/3, which form a heterodimeric complex with the common mediator Smad (co-Smad or Smad4) and translocate to the nucleus [18,20]. Once in the nucleus, the Smad complex acts as a transcription factor able to bind chromatin and modulate its structure. To achieve a high binding affinity for the Smad-binding elements (SBE) in the TGF-*β* target gene promoters, the Smad complex associates with other transcription factors [22,23]. Various families of transcription factors, such as forkhead, homeobox, zinc finger, AP1, Ets and basic helix-loop-helix, are Smad partners [23]. Moreover, the Smad complex recruits co-activators, such as p300 and CREB binding protein, or co-repressors, such as retinoblastoma-like 1 protein, to regulate gene transcription [18,20,23]. Therefore, while Smad proteins are intrinsically transcriptional activators, the transcriptional outcome of their target genes often depends on the transcriptional partners associated with Smads [24].

More recently, a novel arm of TGF-*β* signaling has been discovered in which ALK5 activates the R-Smads, Smad1/5, leading to TGF-*β*-induced anchorage-independent growth and cell migration [25,26]. Furthermore, TGF-*β* can alternatively activate the R-Smads, Smad1/5/8 via the T*β*RI ALK1, which is mainly expressed by endothelial cells [27]. In fact, TGF-*β*/ALK1 signaling potentiates and TGF-*β*/ALK5 signaling inhibits endothelial cell proliferation and migration [28,29].

### 2.3. Smad-Independent Signaling

In addition to the Smad-mediated signaling, TGF*β* can also activate Smad-independent signaling pathways through the interaction and association with alternative mediator proteins [30].

TGF-*β* can induce mitogen activated protein (MAP) kinase signaling, including extracellular signal regulated kinases (Erk1 and 2), p38 and c-Jun amino-terminal kinase (JNK) MAP kinases. The activation of Erk MAP kinase requires the recruitment and phosphorylation of the adaptor protein Shc, which will in turn associate with the adaptor protein Grb2 and the GTP exchange factor SOS [31]. This protein complex activates Ras to its GTP-bound form, and the kinase cascade consisting of c-Raf, MEK1 or MEK2, and Erk1 or Erk2. TGF-*β* also induces activation of p38 and JNK MAP kinase pathway through the tumor necrosis factor (TNF) receptor-associated factor 6 (TRAF6) and TAK1. TRAF6 interacts with the TGF-*β* receptor complex and auto-ubiquitylates and become active. Active TRAF6 associates with TAK1, causing poly-ubiquitylation and phosphorylation of TAK1. Active TAK in turn activate p38 MAP kinase and JNK [32,33]. Furthermore, TGF-*β* receptor complexes interact with the polarity protein Par6 and the tight junction protein occludin at epithelial cell junctions. Here, Par6 is phosphorylated by the receptor complex, and associates with Smurf1. The Par6-Smurf1 complex confers ubiquitylation of RhoA and the consequent dissociation of tight junctions. The interaction of occludin with T*β*RI is required for the localization of TbRI to tight junctions, a prerequisite for efficient TGF-*β*-induced dissolution of tight junctions during epithelial-mesenchymal transition [34]. Thus, the dynamic combination of canonical and non-canonical signaling cascades is responsible for the cellular responses to TGF*β* signaling.

### 2.4. TGF-*β* Signaling and Epithelial-Mesenchymal Transition (EMT)

TGF-*β* acts as a common and potent inducer of EMT via Smad-dependent and independent activation of the expression of the EMT transcription factors Snail, Slug, ZEB1 and 2, and Twist [35,36]. The Smad3/4 complex directly binds the regulatory portion of the promoter of Snail, inducing its transcription. Subsequently a Smad3/4/Snail complex is formed that binds the regulatory promoter sequences of genes encoding for E-cadherin and occludin, leading to repression of their expression [37]. Smad signaling also increases the expression of ZEB transcription factors, which repress miR-200 family expression, further increasing ZEB protein levels and EMT [38]. TGF-*β* also regulates the expression of MMP2 and 9, and ECM components (*i.e.* fibronectin and collagens) [39]. Moreover, TGF-*β* can activate EMT transcription factor expression via alternative splicing [40].

EMT is also controlled by a group of microRNAs that define changes in cytoskeleton reorganization and epithetlial polarity, and it is directly activated in response to TGF-*β* via the Smad/RhoA pathway [41].

Smad-independent TGF-*β* signaling pathways, such as the PI3K/Akt/mTOR pathway, result in increased protein synthesis and cell motility and invasion during EMT. TGF-*β* also induces EMT through ubiquitylation and sumoylation. Smad3/4 complex regulates the expression of HDM2, increasing the ubiquitylation and degradation of p53, inducing EMT progression [42]. TGF-*β* signaling downregulates the expression of the SUMO E3 ligase PIAS1, reducing the levels of sumoylated SnoN, and antagonist of TGF-*β* mediated EMT [43].

## 3. TGF-*β* IN BREAST CANCER PROGRESSION

TGF-*β* plays an essential role in maintaining homeostasis in many tissues through its ability to induce cell cycle arrest, differentiation and apoptosis, thereby preventing uncontrolled proliferation of epithelial, endothelial and hematopoietic cells. It is considered the most potent growth inhibitor for epithelial, hematopoietic and immune cells, because of its ability to induce cell cycle arrest, differentiation and apoptosis, preventing uncontrolled proliferation of these cells [44,45]. However, in many cancers TGF-*β* signaling is compromised, because of the genetic loss of some of the pathway components or due to the downstream influence of other signaling pathways. Hence, these tumors become refractory to TGF-*β* growth inhibition and the pro-tumorigenic actions of TGF-*β* may prevail, including immunosuppression, induction of angiogenesis and promotion of the EMT, thus facilitating cancer migration and invasion (reviewed in [27,46,47]).

### 3.1. Dual Role of TGF-*β* in Breast Cancer Progression

Transgenic mouse models have been particularly informative to understand the roles of TGF-*β* in mammary gland development and tumor progression. Three independent studies tried to inhibit TGF-*β* signaling in mammary tissue by using the mammary gland selective mouse mammary tumor virus (MMTV) promoter to drive the expression of either a soluble T*β*RII:Fc fusion protein [48], a dominant negative T*β*RII (DNT*β*RII) [49] or full length T*β*RII antisense [50]. A proliferative mammary gland phenotype was observed in all models, consistent with the homeostatic role of TGF-*β*, while spontaneous mammary tumors developed only in the DNT*β*RII transgenic model, but these were mostly carcinoma *in situ*, and arose after a prolonged latency [49].

In two additional studies, transgenic mice expressing the activated neu gene in the mammary gland were crossed with strains that expressed either active TGF-*β*1 or constitutively active T*β*RI/ALK5 [51,52]. A markedly delayed primary tumor development was observed in both cases, and tumor growth was slower than in neu single transgenic mice, underpinning a tumor suppressing role for TGF-*β* [51,52]. Nevertheless, the carcinomas that did arise in the double transgenic models were more invasive and aggressive than those occurring in MMTV-neu single transgenics. Zakharchenko *et al*. identified two novel TGF*β*-dependent phosphorylation sites of 14-3-3*σ*, Ser69 and Ser74. They found 14-3-3*σ* phosphorylation to be a feed-forward mechanism in TGF*β*/Smad3-dependent transcription, therefore TGF*β*-dependent 14-3-3*σ* phosphorylation may facilitate the formation of the protein complexes, including Smad3 and p53, at the Smad3-specific CAGA element. Also, breast tumor mouse xenograft and radiobiological assays suggested the involvement of phosphorylation of 14-3-3*σ* at Ser69 and Ser74 in the cancer progenitor population regulation and the radioresistance in breast cancer MCF7 cells. This study suggests that TGF*β*-dependent phosphorylation of 14-3-3*σ* may play a role in the maintenance of cancer stem cells [53]. Recently, it was demonstrated that TGF-*β*1 down-regulated the junction adhesion molecule A (JAM-A) expression via its effects on both the transcriptional and post-translational regulations of JAM-A thus attenuating cell adhesion and promoting cell invasion and that the effect of TGF-*β* was achieved via the activation of Smads [54]. Also, a recent study identifies miR-155-mediated loss of C/EBP*β* as the mechanism that shifts TGF-*β* response in breast cancer from growth inhibition to EMT, invasion and metastasis, promoting breast cancer progression. C/EBP*β* seems to work as a transcriptional activator of genes encoding the epithelial junction proteins E-cadherin and coxsackie virus and adenovirus receptor [55]. These and other [56,57] studies have provided strong support for a tumor-suppressive role for epithelial TGF-*β* signaling in mammary gland tumorigenesis. However, while TGF-*β* may inhibit the growth of mammary tumors in the early stages, it also appears, in these models, to enhance the metastatic potential of those carcinomas that are able to overcome the TGF-*β*-dependent growth suppression and develop.

### 3.2. TGF-*β* Expression Levels in Human Breast Cancer

When the TGF-*β* suppressive effects are lost, TGF-*β* overproduction is commonly observed in many solid tumors. TGF-*β* expression level is often higher in breast cancer compared to normal mammary gland tissue and it appears to increase in the advanced stages of tumor progression [58–60]. Moreover, TGF-*β* expression levels correlate with prognosis and angiogenesis in breast cancer patients [61]. Plasma TGF-*β*1 expression has also been found increased in breast cancer patients, and its level correlates with disease stage [62–65]. Plasma TGF-*β*1 levels have a prognostic value also after tumor resection: patients whose plasma TGF-*β*1 levels normalized after surgery had a better prognosis than those patients with persistently elevated levels, who had higher risk of lymph node metastases and disease progression [64]. These data may suggest an important causal role for TGF-*β* in metastases and disease progression.

Plasma TGF-*β*1 levels have also been determined in 49 bone metastasis patients, including 23 breast cancer patients, and were reported to be elevated in more than half of the cancer patients and positively correlated with TGF-*β* signaling related markers, including parathyroid thyroid hormone-related peptide (PTHrP) and interleukin 10(IL-10) [66]. A recent study shows that elevated circulating levels of TGF-*β* and CXCL1 are associated with a poor prognosis, and higher detection of circulating tumor cells and propensity of these cells to seed lung metastases in patients with breast cancer [67].

TGF-*β* plasma levels may be indicative of TGF-*β*-dependent metastatic disease and may be useful biomarkers to predict the success of treatment with TGF-*β* antagonists in metastatic disease. Ongoing clinical trials are trying to answer these questions. In addition, a highly significant association between T*β*RII expression and reduced survival has been detected in patients bearing estrogen receptor negative breast cancer [68]. Richardsen *et al*. recently published immunohistochemical data from 38 cancer patients: high TGF-*β* levels can be detected in both primary and metastatic tumors and high stromal TGF-*β* expression is associated with increased mortality [69].

Due to TGF-*β* dual nature in breast cancer, its use as single tumor marker that might distinguish patients with high risk of metastases is unlikely. Molecules involved in the TGF-*β* downstream signaling play an important role in determining TGF-*β* prognostic implication, as shown in a retrospective cohort study in patients with invasive non-metastatic breast cancer. High expression of Smad4 showed a trend for better prognosis while high expression of pphosphorylated-Smad2 was associated with poor prognosis [70].

### 3.3. TGF-*β* and Breast Cancer Stem Cells

An increasing body of basic and clinical studies have provided evidence of self-renewing, stem/progenitor-like cells within solid tumors, which have also been referred to as cancer stem cells (CSCs) [71–77]. CSCs are belived to constitute a small minority of neoplastic cells within a given tumor and are defined by their ability to propagate a tumor and potentially seed new metastases [74]. The concept of CSCs remarks the importance of targeting the correct cell population in cancer therapy in order to obtain better results in terms of survival and tumor relapse. Conventional treatments aim to eliminate the rapidly dividing cells in a tumor, leaving space and time to the slower proliferating, less differentiated CSCs to repopulate the tumor [78].

By sorting breast cancer cells for a normal mammary stem cell phenotype (CD44+/CD24^−^/low), Al-Hajj *et al*. was the first to isolate the breast CSC fraction [71]. More recently, Shipitsin *et al*. demonstrated that vimentin, connective tissue growth factor (CTGF), PAI-1, osteonectin, as well as T*β*RII were coexpressed with CD44 [79]. In fact, many of the genes actively transcribed by CD44+ cells were associated with a mesenchymal phenotype and many were known TGF-*β* target genes. They were also able to associate this gene signature with poor prognosis [79]. Mani *et al*. demonstrated that EMT generates cells with properties of stem cells [80]. TGF-*β*-induced EMT in immortalized human mammary epithetlial cells (HMEC) was associated with the acquisition of the CD44^+^/CD24^−^/low phenotype and mesenchymal traits, and increased ability to form mammospheres, a property associated with mammary epithelial stem cells [81]. Paracrine and autocrine signals induce and maintain mesenchymal and stem cell states in the breast [81]. In addition, forcing EMT by overexpressing the EMT transcription factors (and TGF-*β* target genes) SNAIL1 or TWIST also resulted in a CD44^+^/CD24^−^/low phenotype that displayed enhanced tumorigenic potential when injected in mice. In a recent study, the loss of DUSP4, a downstream molecule in the TGF-*β* apoptosis signaling pathway, was shown to increase mammosphere formation and the expression of the CSC-promoting cytokines IL-6 and IL-8 in a mo- del of basal-like breast cancer (BLBC). These effects were caused in part by loss of control of the MEK and JNK pathways and involved downstream activation of the ETS-1 and c-JUN transcription factors. Enforced expression of DUSP4 reduced the CD44^+^/CD24^−^ population in multiple BLBC cell lines in a MEK-dependent manner, limiting tumor formation, again underpinning the dual role of TGF-*β* in breast cancer [82]. Taken together, these studies provide evidence that TGF-*β* is important in regulating the dynamics of cancer cell populations by favoring CSC selfrenewal and inhibiting the commitment to differentiation.

## 4. TGF-*β*, BREAST CANCER AND BONE METASTASIS

### 4.1. Normal Bone Physiology

Bone is primarily made of type I collagen that is mineralized by hydroxyapatite. By weight, bone is about 60% mineral, 10% water and 30% organic matrix. Mineral are made of hydroxyapatite crystals a naturally occurring calcium phosphate. The organic matrix is 98% type I collagen and 2% noncollagenous protein. The noncollagenous proteins include growth factors and cytokines, and extracellular matrix proteins such as osteonectin, osteopontin, bone sialoprotein, osteocalcin and proteoglycans. Although noncollangenous components make small contributions to the overall bone volume it represents major contributions to its biologic function. Growth factors and cytokines such as transforming growth factor-*β* (TGF-*β*), bone morphogenetic proteins (BMPs) osteoprotegerin (OPG), insulin-like growth factor (IGF), interferon-*γ*, the tumor necrosis factors (TNFs) and the interleukins (ILs), are present in very small quantities in bone matrix but have critical effects regulating bone cell differentiation, activation and growth.

The cellular component of bone that is associated with the bone homeostasis the bone resorbing osteoclasts, the bone forming osteoblasts and the cell embedded in the bone matrix, the osteocytes. Osteoclasts arise from hematopoietic progenitors that also give rise to monocyte/macrophages lineage. The osteoclast precursor cells are recruited to the bone surface where they fuse to form large multinucleated cell. The interaction of the osteoclast precursors with the stromal cell and the osteoblasts, in the presence of several intermediary factors such as PTH, Vitamin-D, IL-6, IL-11 and PGE2, induces the central mediator of osteoclast differentiation the RANKL. RANKL or receptor activator of NFκB is a membrane bound member of the TNF receptor family expressed at the osteoblast surface. RANKL binds to its receptor, RANK, which is expressed on the surface of osteoclast precursor. The interaction of the RANKL with its recaptor RANK stimulates the osteoclast differentiation. RANKL/RANK knockout mice develop severe osteopetrosis due to total lack of osteoclast [83,84]. OPG is another TNF superfamliy member, it a soluble factor that act as a decoy receptor for RANKL. When RANKL binds to OPG it prevents the interaction of RANKL with RANK and inhibits osteoclast activation. OPG knockout mice develop osteoporosis as a result of increase in number of osteoclast.

The bone forming osteoblasts are derived from mesenchymal stem cells, pluripotent cells that can differentiate into a variety of cell types including myoblasts, adipocytes, chondrocytes, osteoblasts, and osteocytes.

Osteoblasts are the bone cells that secrete the organic matrix within which several growth factors are embedded. Runx2 and Osterix are two transcription factors that are required for osteoblast formation and differentiation. The regulatory activity of these central osteoblast regulators is modified by cofactors including members of the Dlx (distaless), Msx, and Hox homeodomain gene families and downstream signal transduction mediators such as the TGF-*β* superfamily-related SMADs. As active osteoblasts produce bone matrix (osteoid), they become embedded into their own product and at this stage it is called an osteocyte.

Osteocytes make up to 95% of all bone cells. Osteocytes create an interconnected network in bone allowing for intercellular communications between each other and the surface-lining osteoblasts [85]. Osteocyte senses mechanical load through their canalicular processes and initiate a series of biochemical signaling events that coordinate and influence the activity of osteoprogenitor cell, osteoblasts and osteoclasts, which in turn respond by remodeling bone mass [86,87]. Sclerostin, a secreted protein expressed by osteocytes, responds to mechanical load. Sclerostin plays a central role in the anabolic response of bone to mechanical. Mechanical loads repress Sclerostin mRNA and protein expression thus, releasing the inhibition on new bone synthesis [88].

Bone is a dynamic structure and adult bone is continuously remodeled by the coordinated activities of bone-resorbing osteoclasts and bone-forming osteoblasts [89]. The continuous remodeling process is necessary to replace defective bone as well as to release calcium for various metabolic processes It is the balance between the osteoclasts and osteoblast activity is what keep constant bone mass and the disruption of this balance will result is significant pathological condition such as osteoporosis or osteopetrosis.

### 4.2. TGF-*β* and Bone Interaction

The involvement of growth factors, cytokines and cell adhesion molecules in the remodeling process is what makes bone an attractive site for cancer metastases.

TGF-*β*1 is one of the most abundant growth factors in bone matrix [90]. It is an essential factor for bone remodeling and can affect both bone formation and resorption. The effects of TGF-*β* on osteoblast, osteoclasts and bone remodeling are complex and are both spatial and temporal-dependent [91]. Bone is resorbed by osteoclasts and when the resorption process is completed, a reversal period follows after which osteoblasts deposit new bone matrix to fill the resorption cavity, a process known as coupling. The newly deposited collagenous matrix will be mineralized following a resting phase.

Evidence is accumulating that TGF-*β* is a key mediator in coupling bone resorption to bone formation [92]. Osteoblasts secrete TGF-*β*, where it is embedded into the mineralized bone matrix [93,94]. TGF-*β* is stored in the bone matrix in a latent form. Upon bone resorption by osteoclasts, TGF-*β* is release and activated which in turn activates the proliferation of osteoblast precursor which migrates to the sites of bone resorption [95]. The exposed bone mineral matrix and release of osteotropic factors, such as bone morphogenetic proteins (BMPs), insulin growth factor (IGF)-I and -II, and platelet derived growth factor (PDGF), may then promote differentiation of the osteoblast precursor to osteoblasts [96]. It was shown that TGF-*β* block osteoblast differentiation and bone mineralization in later phases of osteoblastic differentiation [97]. In a coculture of osteoclast precursors with osteoblast and stromal cells, TGF-*β* was shown to inhibit resorption factors such as RANKL and M-CSF while activating the expression of osteoclast inhibitors such as OPG [98,99].

TGF-*β* is a major regulator of osteoclast function either directly or indirectly through its effect on osteoblast. The importance of TGF-*β* on osteoclastogenesis is clear but the exact mechanism is unclear. During bone resorption osteoclasts secrete cathepsins, which proteolytically release activate TGF-*β* from the latent complex [100,101] and because osteoclast express both TGF-*β* and its receptors they can respond directly to TGF-*β* signaling. TGF-*β* can inhibit the recruitment of osteoclast precursors in fetal bone culture but enhances bone resorption by stimulating proliferation and differentiation of osteoclast precursors. TGF-*β* also enhances osteoblast lineage RANKL expression, thus promoting osteoclast precursor recruitment [102].

It has been recently reported by Nguyen, *et al.*, that mechanical load rapidly represses the net activity of the TGF-*β* pathway in osteocytes. This result in reduced phosphorylation and activity Smad2 and Smad3 thus compromises the anabolic response of bone to mechanical load, demonstrating that the mechanosensitive regulation of TGF-*β* signaling is essential for load-induced bone formation [103].

### 4.3. TGF-*β* and Osteolityc Bone Metastases

Bone is a common site of dissemination for breast cancer. The bone microenvironment consists of a rich store of multiple growth factors including TGF-*β*. The metaphyseal bone, which is predominantly composed of trabecular bone and is highly vascular, appears to be the preferred site for bone metastases. Bone metastases develop in about 70% of patients with advanced breast cancer. This is usually a late complication of cancer that can lead to debilitating skeletal related events such as pain, fractures, hypercalcemia and nerve compression which reduce the patient’s quality of life [5,6].

Metastasis to bone is a complete multistep process of events that involves an interaction between the tumor and the host cells. This multifaceted process consists of a series of steps whereby cancer cells detach from the primary tumor, enter into the circulation, disseminate to distal bone sinusoids, enter the bone marrow by extravasation, adapt to the new microenvironment, and eventually grow into lethal tumor which colonies the bone [104].

In bone metastasis biopsies from patients with breast cancer, 75% show positive nuclear staining for phosphorylated-Smad2, as seen on histological sections, indicating an active TGF-*β* signaling [105].

It has been well established in the literature that TGF-*β* signaling pathway play an important role for the development of bone metastases. Several studies uncovered a complicated and context dependent picture regarding the function and utility of TGF-*β*. In an animal model of breast cancer bone metastases, MDA-231 cells were transduced with a retroviral vector expressing a reporter gene under the control of a TGF-*β*-sensitive promoter. In this experiment, it was demonstrated that using this reporter, active TGF-*β*-Smad signaling specifically in the bone was detected and that Knockdown of Smad4 expression in breast cancer cells reduced the growth of bone metastases [105]. In another bone metastases model the expression of inhibitory Smad7 dramatically decrease bone metastases in 1205Lu melanoma models, further implicating role of TGF-*β* in the bone metastases process [106].

**Figure 1:**
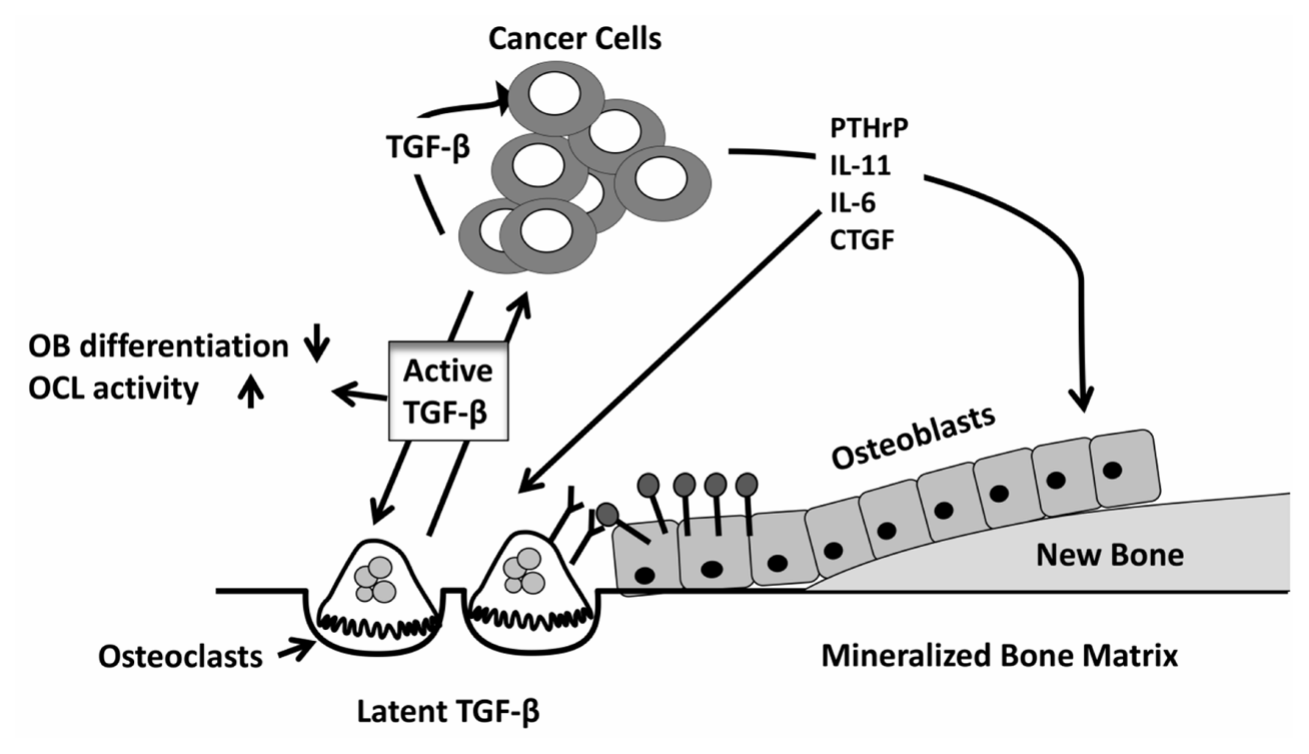
Breast cancer bone metastases. When active TGF-*β* is released from the bone matrix upon bone resorption by osteoclasts it acts on breast cancer cells to stimulate the production of osteolytic factors, such as parathyroid hormone-related protein (PTHrP), connective tissue growth factor (CTGF) and interleukin-(IL) 6 and −11. These factors increase the RANKL/OPG expression ratio in osteoblasts, which bind to the RANK receptors expressed on osteoclasts and activate osteoclastogenesis. TGF-*β* can directly stimulate osteoclast activity and inhibiting osteoblast differentiation thus, TGF-*β* can stimulate tumor growth.

TGF-*β* is able to promote and aggravate bone metastases through specific gene inductions. The TGF-*β*-Smad signaling pathway induces the production of proosteolytic factors, such as interleukin 11 (IL11), connective tissue growth factor (CTGF), matrix metalloproteinase-1 (MMP-1), CXCR4 and parathyroid hormone-related protein (PTHrP) [107]. PTHrP is widely expressed in many tissues and shares sequence homology with PTH. It is known to be expressed in most primary breast cancers tumors as well as in bone metastases. PTHrP plays a major role in the development of the osteolytic lesions and is considered to be responsible for the humoral hypercalcemia of malignancy [108]. In a large prospective study it was demonstrated that PTHrP expression in primary breast cancer was significantly associated with less bone metastases [109–111]. This study could give the explanation of the observed increase in PTHrP expression in breast cancer bone metastases, which is, it is the release of TGF-*β* from the bone matrix after bone resorption is what causes the cancer cells to express PTHrP and not the tumor cells that colonized the bone intrinsically express higher PTHrP level. In mouse model of bone metastases, it was first demonstrated by Yin *et al*. that blocking TGF-*β* signaling by stably transfecting a dominant negative T*β*RII (DNT*β*RII), in MDA-231 breast cancer cells, inhibited TGF-*β*-induced expression of PTHrP production in tumor cells. This is in return suppressed the development of osteolytic lesion area [11]. In another study, I was reported that stable overexpression of dominant-negative Smad 2, 3 and 4 in MDA-231 breast cancer cells resulted in decrease in PTHrP production [112]. TGF-*β*-induced PTHrP stimulated the production of RANKL and downregulating OPG thus inducing osteoclast differentiation and activation and promoting bone metastases [113]. IL-11 and CTGF both is pro-osteolytic gene. IL-11 stimulates the expression of osteoclastogenic factors RANKL and GM-CSF in osteoblasts and stimulating bone resorption. CTGF is an extracellular mediator of invasion and angiogenesis. Both, IL-11 and CTGF are shown to be directly regulated by TGF-*β* via the canonical TGF-*β*/Smad pathway in metastatic cells [10] (Figure 1).

Hypoxia is observed in most solid tumors due to low oxygen concentration [114]. A major mechanism mediating adaptive hypoxia is the regulation of transcription by hypoxia-inducible factor 1 (HIF-1). The bone microenvironment is known to be hypoxic with an oxygen level between 1% and 7% [114]. The enhanced expression and activation of (HIFs) frequently occur during cancer progression and is associated with their acquisition of a more malignant behavior, and hypoxic cells are also considered to be resistant to most anticancer drugs partially due to upregulation of genes involved in drug resistance [115–117].

It was previously shown that HIF-1*α* promote formation of osteolytic bone metastases from breast cancer cell, MDA-MB-231, and that was through stimulating angiogenesis, osteoclastogenesis and inhibition of differentiation of osteoblasts [118]. Multiple interactions exist between hypoxia and TGF-*β* biology. HIF-1a degradation is inhibited by TGF-*β* causing it stabilization. *In vitro* data showed an additive responses to HIF-1*α* and TGF-*β* in the induction of vascular endothelial growth factor (VEGF) and CXCR4 [119,120]. In an animal model of breast cancer bone metastases, inhibition of HIF-1*α* or TGF-*β* by either knock down or DNT*β*RII causes significant reduction in metastases formation with no additive effect when blocked simultaneously [120]. A combined pharmacological inhibition of both HIF-1*α* and TGF-*β*, which targets both cancer cells and bone microenvironment had an additive effect more than either treatments alone indicating that hypoxia and TGF-*β* signaling drive in parallel tumor bone metastases and that pharmacological inhibitors, by acting on both tumor cells and the bone microenvironment, can additively decrease tumor burden [120].

## 5. TGF-*β* AS THERAPEUTIC TARGET

As a result of its wide variety of effects, TGF-*β* signaling provides many therapeutic opportunities for the treatment of disease. The major classes of TGF-*β* inhibitors that have been investigated include: (1) ligand traps, including monoclonal neutralizing TGF-*β* antibodies and soluble decoy receptor proteins; (2) receptor kinase inhibitors, which inhibit T*β*RI/ALK5 (and T*β*RII) kinase activity and prevent the downstream signaling; (3) antisense oligonucleotides, which inhibit TGF-*β* expression at the transcriptional/translational level.

### 5.1. Neutralizing Antibodies and Soluble Decoy Receptor Proteins

TGF-*β* levels and downstream signaling is often increased during cancer progression and is correlated with aggressiveness and grade/stage of the tumor [46,50,121, 122]. Reducing the amount of active TGF-*β* signaling is achieved either via TGF-*β* ligand trap, which uses a soluble decoy receptor comprised of the T*β*RII or T*β*RIII ectodomain, or via neutralizing TGF-*β* antibodies. Neutralizing antibodies have been developed to target individual ligands as well as all three TGF-*β* isomers (pan-neutralizing antibody). The pan-neutralizing mouse monoclonal antibodies, 1D11 and 2G7, bind and reduce biological activity of all three TGF-*β* isoforms and have demonstrated therapeutic potential in mouse tumor models. Treatment of mice harboring MCF-7 breast cancer cells totally abrogated tumor growth [123] and suppressed growth of established MDA-MB-231 sub-cutaneous tumors and lung metastases in athymic mice [124]. Similarly, treatment of mice with 1D 11 following orthotopic injections of 4T1 breast cancer cells suppressed metastasis to lungs [125–127]. 1D11 has also been shown to reduce skeletal tumor burden and osteolytic bone lesions and increase bone volume caused by MDA-MB-231 cells [128].

Another approach to prevent binding of TGF-*β* to its receptors is the use of recombinant Fc-fusion proteins containing the soluble ectodomains of T*β*RII or T*β*RIII. These biologically active compounds have been shown to reduce lung and breast cancer metastases in animal models [31,121,129,130].

### 5.2. Antisense Oligonucleotides (ASO’s)

Antisense oligonucleotides (ASO’s) reduce expression of specific target proteins. ASO’s are single-stranded poly-nucleotide molecules 13 – 25 nucleotides in length that are designed to hybridize to complementary RNA sequences. ASO’s inhibit mRNA function and protein synthesis via modulation of splicing and inhibition of translation [131,132]. ASO’s against TGF-*β* reduce the bioavailability of active ligands in the local tumor microenvironment. To address the role of autocrine TGF-*β* in metastasis formation, Muraoka-Cook *et al*. used an orthotopic model of PyMT mammary tumors [122]. While PyMT tumors overexpressing TGF-*β* resulted in increased metastasis and survival, overexpression of a TGF-*β* ASO reduced metastasis and survival [122].

### 5.3. Small Molecule Receptor Kinase Inhibitors

TGF-*β* receptor kinase inhibitors are small molecule inhibitors that act via ATP-competitive inhibition of the kinase catalytic activity of T*β*RI/ALK5. There are advantages to the development and scalability of small molecule inhibitors but the potential lack of selectivity of kinase inhibitors is problematic. Currently, all known small molecule T*β*R1/ALK5 inhibitors described display equipotent inhibition against ALK4 kinase activity and less inhibition against ALK7 [133–137].

Inhibitors of T*β*RI/ALK5 have been extensively studied including: SB-431542 [136] (GlaxoSmithKline), Ki26894 (Kirin Brewery Company) [138], LY364947 (Eli Lilly & Co.), and SD-208 and SD-093 (Scios Inc). Each of these compounds blocks receptor kinase activity and inhibits proliferation, invasion or metastasis of tumor cells in animal models [134–136]. In a xenograft model of intracardiac inoculated MDA-MB-231 human breast cancer cells for example, SD-208 significantly inhibited the size of osteolytic lesions, bone metastatic growth and survival. Furthermore, SD-208 treatment in mice with already established bone metastases inhibited further tumor growth and formation of osteolytic lesions [120]. The same treatment was shown to increase bone mass in non-tumor model which could be of mutual bebefits for cancer patients reduced osteolytic lesion and increasing bone mass [139].

### 5.4. Other Molecules that Antagonize TGF-*β*

Additional biologically active molecules that inactivate TGF-*β* or its signaling have also been described. The natural product derivative halofuginone (Hfg) recently completed phase II clinical trials for the treatment of sarcoma [140]. We recently published that Hfg inhibits TGF-*β* signaling *in vitro* in several cell types, and that systemic daily treatment of Hfg in mice significantly inhibits the formation of osteolytic lesions and bone metastases after intracardiac inoculation melanoma1205Lu [141]. Although the exact mechanism remains to be investigated, Hfg treatment represents a novel agent to inhibit TGF-*β* signaling in bone metastasis.

### 5.5. Combination Therapy

An attractive approach to increase treatment efficacy for patients with bone metastases is to combine treatments that antagonize the effects of TGF-*β* with other therapies. For example, targeting TGF-*β* signaling can enhance the therapeutic efficacy of various cytotoxic agents as was recently shown for rapamycin [142] and doxorubicin [143,144]. Studies in our laboratory show that SD-208 dosed in combination with an inhibitor of bone resorption, zoledronic acid, reduces the progression of established osteolytic metastases from breast cancer more effectively than either therapy alone [145]. Using the same bone metastasis model of intracardiac inoculation of MDA-MB-231 breast cancer cells, we tested the effects of a combined treatment of SD-208 and 2-methoxyestradiol, an inhibitor of HIF-1*α*, the key mediator of hypoxia. Combined treatment with these agents reduces osteolytic lesions, tumor burden and improves survival of mice more effectively than either treatment alone [120].

### 5.6. Risks, Limitations and Opportunities

As a result of its biological importance and wide variety of effect, blockade of TGF-*β* or its signaling provides intriguing therapeutic opportunities for the treatment of many different disease indications. However, potent and/or chronic inhibition of this wide-spread biologically important molecule may also potentially result in a variety of undesirable side effects.

Drug delivery challenges must be overcome for ASO-based therapies and large biological (neutralizing antibodies). The generation of small molecules such as TGF-*β* receptor kinase inhibitors overcomes the necessity of injectable delivery, loss of efficacy due to neutralizing antibody generation and/or tissue penetration issues commonly observed with biologic-based agents and most are suitable for oral dosing [12]. However, TGF-*β* recaptor kinase inhibitors used so far are less selective than the current TGF-*β* ASO’s or biologic-based TGF-*β*-directed therapies.

Other promising approaches to overcome off-target tissue toxicity and poor drug exposure to tumor cells in bone metastatic disease are the use of bisphosphonate to deliver therapeutics directly to bone. One approach is a bisphosphonate-coated liposome, which may be useful as a targeting device to sites of high bone turnover, includeing sites with bone metastatic disease [146]. Another possibility is targeting anti-TGF-*β* therapies via conjugation of small molecule inhibitors to bisphosphonates. Potentially, these bone-targeted strategies may allow for a more prolonged local exposure to higher concentrations of the compounds thereby enhancing therapeutic efficacy and minimizing systemic side effects. Additionally, these bioactive compounds of interest could be delivered to bone metastatic sites in combination with other anticancer agents with synergistic or mechanistic action.

## 6. CONCLUSION

TGF-*β* is a pluripotent cytokine with a prominent role in breast cancer progression and bone metastasis. TGF-*β* is a central mediator in driving a feed-forward vicious cycle of tumor growth in bone. Thus much effort has been placed on development of agents to inhibit TGF-*β* activiity. Currently, three therapeutic modalities targeting TGF-*β* have been pursued and are presently being tested in clinical trials in cancer patients (incl. bone metastatses): TGF-*β* antibodies, TGF-*β* receptor kinase inhibitors and TGF-*β* antisense oligonucleotides. TGF-*β* has many other functions in normal physiology, and may also act as a tumor suppressor in certain malignancies. Therefore concerns will remain that long-term blockade of this pathway may have other off-target effects. The next decade should reveal new and exciting clinical data that will help determine which TGF-*β* therapeutic strategies are most effective for the treatment of patients.
